# Mental health reform in Australia – unfinished business

**DOI:** 10.1192/bji.2023.19

**Published:** 2023-11

**Authors:** Sebastian Rosenberg, Luis Salvador Carulla, Alan Rosen

**Affiliations:** 1 Senior Lecturer, Brain and Mind Centre, University of Sydney, Camperdown, New South Wales, Australia. Email sebastian.rosenberg@sydney.edu.au; 2 Professor, Health Research Institute, University of Canberra, Canberra, Australian Capital Territory, Australia; 3 Professor, Australian Health Services Research Institute, University of Wollongong, Wollongong, New South Wales, Australia

**Keywords:** Australia, mental health, policy, planning, human rights

## Abstract

Australia was one of the first countries to develop a national mental health strategy. This article reviews the progress of reform, outlining some strengths, weaknesses and prospective challenges.

Australia's National Mental Health Strategy began in 1992. The national mental health reform process could be characterised as diverse and uneven rather than steady or linear.

## Healthcare structure

Responsibility for funding and planning healthcare in Australia is split between the Federal (national) government and eight states and territories. The Federal government is responsible for primary care (about Au$4bn for mental health in 2020–2021), historically focusing on the role of general practitioners (and more recently psychologists). These services are funded by our universal health insurer Medicare, with individual practitioners charging a fee for service. The states and territories each have health budgets, principally directed towards the provision of hospital-based in-patient and out-patient care, including in mental health (about Au$7bn).

Australia deinstitutionalised psychiatric care in the 1990s under the National Mental Health Strategy, although 1500 beds in psychiatric specialist institutions remain, costing Au$600m annually.^1^ Most of the acute in-patient care occurs in 5521 mental health beds located in the psychiatric wards of Australia's general public hospitals. Overall, the rate of mental health beds available per 100 000 population declined from 40.2 in 2011–2012 to 37.1 in 2020–2021. Over the same period, the average length of stay in public hospital mental health acute units reduced from 14.6 days to 13.

Responsibility for secondary mental healthcare, especially in relation to community-based clinical and psychosocial mental health services, is unclear. In the 1980s and 1990s, Australia could reasonably be described as leading the world in the establishment of multidisciplinary community mental health teams but many of these services have been depleted.^2^

## Epidemiology

Over 2 in 5 (44%, or 8.6 million) Australians aged 16–85 experience a mental disorder at some time in their life, with 1 in 5 (21%, or 4.2 million) having experienced a mental disorder in the previous 12 months.^3^ Prevalence rates seem stable, although recent data indicate increasing levels of mental illness among Australia's youth,^4^ a trend common to many countries.^5^

Mental and substance use disorders contribute 13% to Australia's total burden of disease, the fourth highest contributor. They are the second highest contributor to non-fatal burden (24%), behind musculoskeletal conditions.^6^ Mental health's share of total health spending has not changed: 7.3% in 1992–1993 and 2020–2021.^1^ The considerable gap between the level of funding and mental health's share of the burden of disease is one factor inhibiting the nationwide development of Australia's mental health services.

Recent investment in Medicare-subsidised psychology services has seen growth in the number of Australians receiving mental health treatment, from 35% to 46%,^7^ although the outcomes of this treatment are unclear.^8^

In 2021, 3144 Australians died by suicide,^9^ an age-standardised suicide death rate of 12.0 per 100 000 people. Although this rate fluctuates, the 20-year trend is upwards, for both men and women.

## Workforce

The Australian Institute of Health and Welfare reports that in 2020 (during the COVID-19 pandemic), there were 84.4 mental health nurses, 78.4 psychologists and 11.6 psychiatrists working in full-time clinical roles per 100 000 population, an increase from 75.8, 61.8, and 10.1 in each category respectively since 2013. Australia's investment in ‘lived experience’ workers is recent and limited. Nationally, 328.8 full-time paid peer support workers and 103.4 paid carer workers were employed in specialist mental health services. Overall, services and workforce are concentrated in urban areas, with increasing gaps evident elsewhere.^10^

## Legislation and jurisdictional differences

Each state and territory has its own mental health legislation, largely derived from ‘model’ legislation but with some differences, leading to variations in the regulation of involuntary treatment, among other matters.^11^

Each jurisdiction also has separate systems of financing, accountability and data collection, although with national data monitoring, comparable to other federal countries and countries with regional governance and financing, such as Belgium, Italy or Spain. Jurisdictional variation in Australia translates into notable regional differences in care type, availability, equity and access.

## Planning and development

Australia's National Mental Health Strategy was developed partly in response to issues emerging from a national inquiry by the Human Rights and Equal Opportunity Commission, which found egregious examples of poor treatment and care in mental health services. Repeated examples of this kind of statutory inquiry have become a defining feature of mental healthcare in Australia.^12^

The 1993–1998 National Mental Health Plan began a series of five such plans, with the most recent concluding in 2017. No evaluation of the national plans has occurred since 2008. In 2022, rather than agree a new national five-year plan, the Federal government signed individual mental health and suicide prevention plans with each of the states and territories (available at: https://federalfinancialrelations.gov.au/agreements/mental-health-suicide-prevention-agreement).

### The National Disability Insurance Scheme

Australia's mental health system was further fragmented with the 2013 establishment of the National Disability Insurance Scheme (NDIS), a new public insurance scheme designed to meet the needs of Australians with permanent and enduring disabilities, including psychosocial disability.^13^

Around 60 000 Australians with severe and complex psychosocial disability are currently enrolled in the NDIS, out of an estimated 64 000 total eligible. Just under Au$1bn was spent in 2022–2023, a 35% increase from the same time in the previous year. This equates to per capita NDIS spending of nearly $67 000, about 20 times more than is spent on services for people with a mental illness who do not qualify for the NDIS.^14^ This inequity is one of the challenges facing Australia as it grapples with creating a fair and navigable mental health system for everybody.

## Progress

Australia's 1992 National Mental Health Policy set out an ambitious reform agenda focusing on deinstitutionalisation, community mental health, better accountability and the delivery of human rights for people with a mental illness and carers.

Unfortunately, as revealed by repeated statutory and other inquiries, positive rhetoric and benevolent policy intent has often failed to translate into practical improvements in the experiences of mental healthcare felt by patients. Problems with implementation reflect Australia's limited success in developing useful national processes for accountability in mental healthcare. Much data is collected by government service providers, but this has not resulted in effective processes of systemic quality improvement, using validated measures of patient outcomes.^15^ There is little evidence that either the prevalence or severity of mental illness in Australia has diminished over the past 30 years, indeed there is evidence that psychological distress has increased.^16^

Patients’ rights were a central feature of the first National Mental Health Plan in 1993, but it was only in 2022 that national patient and carer mental health organisations were funded nationally.

Although Australian mental health research enjoys a strong international reputation, the National Mental Health Commission has estimated that national investment in mental health research is approximately half what could be expected based on the prevalence and burden of mental ill health among Australians, as compared with other diseases and health problems.

## Where to from here?

Australia's next phase of mental health reform will require action at several levels, spanning not just workforce growth, but also role delineation, service design, outcome measurement, accountability and systemic quality improvement. This work must reflect the regional diversity of Australia. We can draw on new skills and tools in mental health planning and policy development, including the National Mental Health Service Planning Framework^17^ and regional Atlases of Mental Health, revealing regional patterns of mental healthcare for specific age groups (adults, children and adolescents, and older adults), specific services (such as addiction) and jurisdictions.^10^

More sophisticated bottom-up modelling techniques are emerging,^18^ in response to recommendations made by recent inquiries,^15^ on the premise they would permit greater control by local service planners and policymakers, allowing them to consider future risks, shifts and opportunities as well as historical trends. Supporting successful regional implementation will continue to be the key challenge.

Australia's fragmented approach to mental healthcare has led to a confusing and problematic system, subject to repeated inquiry. Significant shortfalls in both mental health service access and quality are evident. Consequent risks to patients and carers remain. The new Federal government, elected in May 2022, has flagged its intention to pursue mental health reform, working in partnership with other governments and with mental health stakeholders (both clinical and psychosocial). The opportunity for positive change is clear.

## Data Availability

Data availability is not applicable to this article as no new data were created or analysed in this study.
